# Comparing characteristics and perspectives of U.S. anesthesiology fellows in training and anesthesiologists in their first year of practice

**DOI:** 10.1186/s12909-023-04890-1

**Published:** 2023-12-15

**Authors:** Emily Toutkoushian, Dandan Chen, Huaping Sun, David O. Warner, Alex Macario, Stacie G. Deiner, Mark T. Keegan

**Affiliations:** 1 The American Board of Anesthesiology, 4200 Six Forks Road, Suite 1100, Raleigh, NC 27609 USA; 2 Department of Anesthesiology and Perioperative Medicine, Mayo Clinic, Rochester, MN USA; 3 Department of Anesthesiology, Perioperative and Pain Medicine, Stanford University, Stanford, CA USA; 4 Department of Anesthesiology, Dartmouth Hitchcock Medical Center, Lebanon, NH USA

**Keywords:** Anesthesiology fellows in training, First-year practicing anesthesiologists, Perceived challenges for the profession of anesthesiology, Anesthesia workforce competition, Value of anesthesiologists

## Abstract

**Background:**

The purpose of this study was to evaluate relationships between demographics, professional characteristics, and perceived challenges facing the specialty of anesthesiology among physicians who entered a fellowship and those who started independent practice immediately after finishing a U.S. anesthesiology residency.

**Methods:**

Anesthesiologists in the year after their residency graduation were invited to take an online survey during the academic years of 2016–2017, 2017–2018, and 2018–2019, with questions about their personal characteristics, the nature of their professional lives, and their perceptions of the greatest challenge facing the profession of anesthesiology.

**Results:**

A total of 884 fellows-in-training and 735 anesthesiologists starting independent practice right after the completion of their residency responded. Fellows were slightly younger (mean = 33.2 vs. 34.0 years old, *p* < 0.001), were more likely to have a spouse who works outside the home (63.9% vs. 57.0%, *p* = 0.002), had fewer children (mean = 0.69 vs. 0.88, *p* < 0.001), worked more hours per week (mean = 56.2 vs. 52.4, *p* < 0.001), and were less likely to report a personal and professional life balance (66.4% vs. 72.3% positive, *p* = 0.005) than direct-entry anesthesiologists. Fellows and direct-entry anesthesiologists identified similar challenges in three broad themes – workforce competition (80.3% and 71.8%), healthcare system changes (30.0% and 37.9%), and personal challenges (6.4% and 8.8%). Employment security issues posed by non-physician anesthesia providers and perceived lack of appreciation of anesthesiologists’ value were commonly cited. Relative weighting of challenge concerns varied between fellows and direct-entry physicians, as well as within these groups based on gender, fellowship subspecialty, location or size of practice, and frequency of supervisory roles.

**Conclusions:**

Anesthesiology fellows and direct-entry anesthesiologists had largely similar demographics and perspectives on the challenges facing anesthesiology in the United States. Group differences found in some demographics and perspectives may reflect different motivations for choosing their professional paths and their diverse professional experiences.

**Supplementary Information:**

The online version contains supplementary material available at 10.1186/s12909-023-04890-1.

## Introduction

In the United States, medical school graduates wishing to pursue a career in anesthesiology typically complete a four-year “residency” in a training program accredited by the Accreditation Council for Graduate Medical Education (ACGME, Chicago, IL). Thereafter, they become eligible for certification by the American Board of Anesthesiology (ABA, Raleigh, NC) through its staged examination system [1]. Such residency programs include a clinical base (CB) year and three clinical anesthesia (CA) years. Residency graduates either continue training in a subspecialty fellowship or go directly to independent practice. Based on enrollment data collected by the ABA and its prior survey of anesthesiology residents’ plans, almost 60% of anesthesiology residency graduates in recent years pursued a fellowship in an anesthesiology subspecialty, which is typically a 1-year commitment [2]. Previous research in other medical specialties has suggested that residents consider multiple factors when making this decision, including their curiosity and passion for the additional skills and knowledge to be gained during the fellowship, their professional goals, whether a fellowship will increase the likelihood of securing employment in a desired setting, financial circumstances, and familial obligations [3–5]. There is limited information [6] about factors that may influence the decision of anesthesiology residents to pursue fellowship training. Understanding the demographics and attitudes of new residency graduates who either choose a fellowship or directly enter independent practice is important because these physicians represent the next generation of anesthesiologists who will drive the future of the specialty. Such information may be useful for residency and fellowship program directors, professional organizations, and workforce planning.

The purpose of this study was to evaluate the relationships between demographics, professional characteristics, and perceived challenges facing the specialty of anesthesiology based on physicians who entered a fellowship immediately after residency (“anesthesiology fellows” [AFs]) and those who entered independent practice immediately after residency (“direct-entry anesthesiologists” [DEs]). The ABA administered voluntary, anonymous, annually repeated cross-sectional surveys to anesthesiologists who started anesthesiology training in an ACGME-accredited residency program in the U.S. between the 2013 and 2018 academic years. Data for this study were collected, for each graduating class, in the year after completion of anesthesiology residency training.

## Methods

The Mayo Clinic Institutional Review Board (Rochester, MN) deemed this study to be exempt from review and provided a waiver of the need for written informed consent from the participants.

The study methodology has been described previously [2, 7, 8]. In summary, all residents were first invited to respond to a clinical anesthesia year 1 [CA-1, also known as post graduate year 2 (PGY-2)] resident survey, and subsequently invited to respond to the surveys on an annual basis through their PGY-5 (i.e., the year after completing anesthesiology residency training). Surveys were administered using the online survey platform QuestionPro (Beaverton, OR). Resident cohorts were defined by their CA-1 year. For the 2013 to 2015 cohorts, the PGY-5 surveys upon which this study was based, were administered in the 2016-17 (spring 2017), 2017-18 (spring 2018), and 2018-19 (spring 2019) academic years, respectively. This analysis included the PGY-5 surveys; other survey results have been published previously [2, 7, 8].

### Survey questionnaire

Two slightly different survey questionnaires were administered to the PGY-5 anesthesiologists based on whether the respondent was in a fellowship program (AF) or had started independent practice (DE). Both survey questionnaires asked a common set of questions about physician demographics, well-being, career planning, and professional life [2, 7, 8], including an open-ended question that asked: “In your opinion, what is the greatest challenge facing the profession of anesthesiology today?” Additionally, several questions specifically applicable to either AFs or DEs were included separately in each version of the PGY-5 questionnaires across all three years. The AF-specific questions focused on the nature of the respondents’ fellowships, their fellowship experiences, and their post-fellowship plans. The DE-specific questions focused on the respondents’ practices, and their roles (if any) in the supervision of residents, certified registered nurse anesthetists (CRNAs), and anesthesiologist assistants (AAs). In total, the AF and DE surveys included 33 and 38 questions, respectively.

### Analytic strategies

The sample inclusion criteria were discussed in detail in previous publications [2, 7, 8]. For each survey, respondents who answered at least 20 questions were included and duplicate surveys were excluded based on demographic variables. Descriptive statistics were used to summarize demographic data and the responses to close-ended questions related to professional lives for both AFs and DEs. To examine possible differences between these two groups, independent-samples t-tests were used to compare values of interval variables, such as age and number of children, and two-proportion z-tests [9] were used to compare the proportions of respondents selecting certain categories of categorical variables, such as yes/no or 5-point agreement scale.

Responses to the open-ended question about the greatest challenge facing the profession of anesthesiology were coded using a systematic thematic analysis process. Iterative, inductive coding was utilized to explore and organize categories and themes that emerged from the data. As a first step, all responses from across the study years were combined and the lead coder identified potential overarching themes by reviewing all responses. Next, a more detailed review of the responses using the preliminary framework generated more themes, and categories within the themes were modified and applied across both the AF and DE groups until saturation of the codes was reached. To validate these codes, a second rater, trained on the code definitions, coded a random sample of 25% of the responses across both groups and all years. The initial agreement between the lead and second raters was 76% for categories and 91% for the three overall themes. The two coders discussed all discrepancies to reach an agreement of 100% across themes and categories for the sample responses — some categories that had enough overlap were collapsed and a few other categories were regrouped into different themes. The lead coder then reviewed all responses again to reflect any coding changes made during the validation process. The coded responses were summarized using counts and percentages of themes and categories. Two-proportion z-tests were conducted to test statistical significance of relevant comparisons at the *p* < 0.05 level, with the Bonferroni correction applied to account for multiple comparisons.

Quantitative analyses were performed using R 4.2.2 (Vienna, Austria); qualitative analysis was conducted in Microsoft Excel with its comment and highlight functions to make notes of the emerging categories and themes.

## Results

A total of 1,619 anesthesiologists (884 out of 2,336 AFs and 735 out of 2,601 DEs) across the 2016 to 2018 academic years completed the PGY-5 surveys. Response rates were 46%, 34%, and 35% for AFs and 29%, 29%, and 27% for DEs for the academic years 2016-17, 2017-18, and 2018-19, respectively.

### Demographic and practice characteristics

AFs were slightly younger, more likely to have a spouse who works outside the home, had fewer children, worked more hours per week, and were less likely to maintain their personal and professional life balance than DEs (Table 1). Other characteristics, including student loan amount, being in a relationship, English as a primary language, having strong social support, and mean number of calls per month, did not differ between the AFs and DEs.

**Table 1:** Comparison of characteristics between anesthesiology fellows and direct-entry anesthesiologists

	Anesthesiology Fellows (AFs)				Direct-Entry Anesthesiologists (DEs)				Statistical significance between AFs and DEs^a^
	2016 (n = 333)	2017 (n = 264)	2018 (n = 287)	All cohorts combined (n = 884)	2016 (n = 254)	2017 (n = 250)	2018 (n = 231)	All cohorts combined (n = 735)	
**Gender (% female)**	44.0%	45.4%	37.3%	42.2%	39.4%	44.4%	43.3%	42.3%	NS^b^
**Mean age**	33.1	33.1	33.5	33.2	34.0	34.0	34.0	34.0	*p* < 0.001
**Mean student loan ($)**	$247,723	$240,418	$262,278	$250,266	$239,785	$237,812	$275,209	$250,247	NS
**In a relationship (% Y)**	77.2%	74.7%	82.6%	78.2%	80.3%	78.8%	77.3%	78.8%	NS
**Working spouse (% Y)**	63.8%	58.4%	69.1%	63.9%	53.5%	61.6%	56.0%	57.0%	*p* = 0.002
**Mean number of children**	0.62	0.78	0.68	0.69	1.06	0.80	0.76	0.88	*p* < 0.001
**Primary language (% English)**	85.6%	77.3%	85.8%	83.2%	83.1%	83.2%	84.6%	83.6%	NS
**Having strong social support** ^c^ **(strongly agree or agree)**	82.9%	81.4%	82.3%	82.3%	81.9%	77.6%	81.1%	80.2%	NS
**Mean number of work hours per week**	57.4	56.8	54.2	56.2	51.6	52.8	53.0	52.4	*p* < 0.001
**Mean number of night calls per month**	4.6	4.2	3.9	4.2	3.8	4.2	4.1	4.0	NS
**Personal and professional life balance** ^d^ **(% strongly agree or agree)**	63.1%	66.6%	70.1%	66.4%	72.9%	73.6%	70.4%	72.3%	*p* = 0.005

^a^ Independent-samples t-tests were used to compare values of interval variables, such as age and number of children, and two-proportion z-tests were used to compare the proportions of respondents selecting certain categories of categorical variables; ^b^ NS = not significant;

^c^ Survey question text: “I have a strong social support system” [Strongly Agree, Agree, Neutral, Disagree, Strongly Disagree];

^d^ Survey question text: “I maintain a balance between my personal and professional life” [Strongly Agree, Agree, Neutral, Disagree, Strongly Disagree]

### Perceived greatest challenge

Perceived greatest challenge expressed in free-text comments fell in three broad themes: (1) workforce competition, (2) healthcare system changes, and (3) personal challenges, with several specific concerns within each theme.

More than three quarters of all respondents indicated that workforce competition was the greatest challenge facing the profession of anesthesiology. Specifically, more than half of respondents identified job encroachment by non-physician anesthesia providers, especially CRNAs (and to a much lesser extent AAs), as the most pressing concern (Table 2). Respondents made statements such as “Losing ground to CRNAs”, “Scope of practice infringement by CRNAs”, and “Our inability to slow down the power and reach of CRNAs” (see more detailed examples in Supplementary Table A). In addition, about one third of respondents felt that those external to the field, including other medical professionals and the public, failed to appreciate the value of anesthesiologists, a problem compounded by a perceived lack of advocacy for the profession. Some mentioned that there was a misconception that anesthesiology was easy and that “mid-level providers” (a general term used for advanced practice nurses and physician assistants) could replace anesthesiologists. This was coupled with unease about apathy or perceived lack of initiative from their anesthesiologist colleagues and lack of defined anesthesiologist roles in the “perioperative surgical home” [10].

Approximately one third of all respondents expressed concerns regarding healthcare system changes affecting the practice of anesthesiology (Table 2). Specifically, one out of six expressed unease about the changing U.S. reimbursement models such as bundling of payments. Typical responses included “Bundled payments [are of concern], especially in the private sector where multiple specialties will be ‘competing’ for their piece of the reimbursement (surgeon, anesthesiologist, hospitalists, etc.)” and “Proving our worth and that our decisions in the OR [operating room] affect outcomes significantly, especially as payments are tied to outcomes”. Other concerns included uncertainty related to politically motivated changes to the healthcare system that were not necessarily of clinical benefit, threats to patient care (e.g., insufficient resources to meet patient needs, production pressure) and the corporatization of practice management by large anesthesia management companies.

Only about one in thirteen respondents expressed concerns regarding personal challenges affecting their practice of anesthesiology. Specifically, about 5% of all respondents expressed concerns about burnout and a lack of personal and professional life balance, and about 2% expressed apprehension about the pressure to pass board certifying exams and maintain required standards.

**Table 2:** Overview of anesthesiology fellows’ and direct-entry anesthesiologists’ perceived challenges to the practice of anesthesiology

Category	Brief definition of category	Count (%) of all responses (n = 1011)	Count (%) of Anesthesiology Fellows’ responses (n = 547)	Count (%) of Direct-Entry Anesthesiologists’ responses (n = 464)
*Theme 1: Workforce Competition and Challenges*		*772 (76.4)*	*439 (80.3)*	*333 (71.8)*
**Competition from non-physician providers**	Non-physician anesthesia providers (such as CRNAs or AAs) encroaching on job security	536 (53.0)	**308 (56.3)**	228 (49.1)*
**External perception of anesthesiologist value**	Lack of recognition of anesthesiologist importance and/or lack of advocacy for the profession	335 (33.1)	189 (34.6)	146 (31.5)
**Apathy from anesthesiologist colleagues**	Challenges related to other anesthesiologists; concerns relating to peer performance or attitudes	44 (4.4)	23 (4.2)	21 (4.5)
**Role of anesthesiologists**	Lack of defined anesthesiologist roles in the perioperative surgical home	32 (3.2)	19 (3.5)	13 (2.8)
*Theme 2: Healthcare System Changes*		*340 (33.6)*	*164 (30.0)*	*176 (37.9)*
**Compensation**	Negative changes to reimbursement or payment models	160 (15.8)	81 (14.8)	79 (17.0)
**Uncertainty of changes in the healthcare system**	Uncertainty due to general or political changes to the overall healthcare system	91 (9.0)	47 (8.6)	44 (9.5)
**Threats to patient care**	Increase in patient acuity; insufficient resources to address patient needs; patient safety and production pressure	85 (8.4)	37 (6.8)	48 (10.3)
**Corporationized management**	Large anesthesia management corporations	78 (7.7)	33 (6.0)	45 (9.7)
*Theme 3: Personal Challenges*		*76 (7.5)*	*35 (6.4)*	*41 (8.8)*
**Psychological pressures**	Psychological pressures including burnout, depression, and family-related pressures	55 (5.4)	24 (4.4)	31 (6.7)
**Meeting the standards**	Contention about ACGME standards, or ABA certifying exams	22 (2.2)	13 (2.4)	9 (1.9)

* = significant difference in proportions comparing AFs and DEs

CRNA = Certified Registered Nurse Anesthetist; AA = Anesthesiologist Assistant; ACGME = Accreditation Council for Graduate Medical Education; ABA = American Board of Anesthesiology

### Perceived challenges according to respondent and practice characteristics

AFs were more likely to articulate concerns about competition from non-physician providers than DEs (Table 2). Regarding differences according to gender, female AFs were more likely to express concerns about competition from non-physician providers than DEs; one respondent stated that “…as a female physician, I am already mistaken for a nurse frequently; the attack on our specialty by anesthetists will only make this worse”. Male DEs were more likely to express concerns about compensation than female AFs (Table 3).

**Table 3:** Perceived challenges to the specialty of anesthesiology by gender for anesthesiology fellows and direct-entry anesthesiologists

	Count (%) of Anesthesiology Fellows’ responses (n = 542)		Count (%) of Direct-Entry Anesthesiologists’ responses (n = 460)	
	Male (n = 351)	Female (n = 191)	Male (n = 307)	Female (n = 153)
**Competition from non-physician providers**	188 (53.6)	**118 (61.8)**	153 (49.8)*	73 (47.7)*
**External perception of anesthesiologist value**	121 (34.5)	66 (34.6)	90 (29.3)	56 (36.6)
**Apathy from anesthesiologist colleagues**	17 (4.8)	6 (3.1)	16 (5.2)	5 (3.3)
**Role of anesthesiologists**	11 (3.1)	8 (4.2)	9 (2.9)	4 (2.6)
**Compensation**	57 (16.2)	21 (11.0)*	**58 (18.9)**	20 (13.1)
**Uncertainty of changes in the healthcare system**	30 (8.5)	16 (8.4)	30 (9.8)	12 (7.8)
**Threats to patient care**	19 (5.4)	18 (9.4)	31 (10.1)	16 (10.5)
**Corporationized management**	25 (7.1)	8 (4.2)	30 (9.8)	14 (9.2)
**Psychological pressures**	17 (4.8)	7 (3.7)	23 (7.5)	8 (5.2)
**Meeting the standards**	8 (2.3)	5 (2.6)	8 (2.6)	1 (0.7)

Note: A bolded and underlined **figure** indicates that the number was statistically significantly different (after Bonferroni correction) from the number(s) with an asterisk in the same row. For example, in the first row, 61.8% in the female fellows’ column was significantly different from 49.8% and 47.7% in the male DEs’ and female DEs’ columns, respectively, but not from 53.6% in the male fellows’ column

The proportion of AFs expressing concerns about competition from non-physician providers, external perception of anesthesiologist value, and threats to patient care varied by fellowship subspecialty (Table 4). Pain medicine fellows were more likely to express concerns about competition from non-physician providers than cardiac anesthesiology fellows, but less likely to express concerns about external perception of anesthesiologist value than critical care medicine and obstetric anesthesiology fellows. Cardiac anesthesiology fellows had more concerns about patient acuity and the availability of resources to care for such patients than critical care medicine fellows.

**Table 4:** Anesthesiology fellows’ perceived challenges to the profession of anesthesiology according to fellowship subspecialties^a^

	Count (%) of Anesthesiology Fellows’ responses (n = 530)				
	Cardiac Anesthesiology (n = 108)	Critical Care Medicine (n = 115)	Obstetric Anesthesiology (n = 32)	Pain Medicine (n = 144)	Pediatric Anesthesiology (n = 131)
**Competition from non-physician providers**	52 (48.1)*	59 (51.3)	19 (59.4)	**93 (64.6)**	72 (55.0)
**External perception of anesthesiologist value**	42 (38.9)	**48 (41.7)**	**15 (46.9)**	37 (25.7)*	42 (32.1)
**Apathy from anesthesiologist colleagues**	5 (4.6)	6 (5.2)	3 (9.4)	6 (4.2)	2 (1.5)
**Role of anesthesiologists**	5 (4.6)	4 (3.5)	0 (0.0)	4 (2.8)	6 (4.6)
**Compensation**	18 (16.7)	19 (16.5)	5 (15.6)	19 (13.2)	20 (15.3)
**Uncertainty of changes in the healthcare system**	6 (5.6)	9 (7.8)	3 (9.4)	14 (9.7)	15 (11.5)
**Threats to patient care**	**11 (10.2)**	3 (2.6)*	3 (9.4)	7 (4.9)	12 (9.2)
**Corporationized management**	8 (7.4)	7 (6.1)	1 (3.1)	9 (6.3)	8 (6.1)
**Psychological pressures**	6 (5.6)	1 (0.9)	2 (6.3)	4 (2.8)	10 (7.6)
**Meeting the standards**	2 (1.9)	3 (2.6)	1 (3.1)	2 (1.4)	4 (3.1)

^a^ 15 practitioners who chose “Regional” and 1 practitioner who chose “Other” were removed from table

Note: A bolded and underlined **figure** indicates that the number was statistically significantly different (after Bonferroni correction) from the number with an asterisk in the same row. For example, in the first row, 64.6% of the Pain Medicine fellows mentioned competition from non-physician providers as the challenge to the profession, which was significantly different from 48.1% of the Cardiac Anesthesiology fellows who mentioned this concern

The DEs’ perceived challenges were analyzed according to their reported job characteristics, including practice size and frequency of performing their own cases versus mainly acting in a supervisory capacity (Table 5), as well as geographical location (Table 6). Practice size was classified into small (fewer than 10 anesthesiologists), medium (10–50 anesthesiologists) and large (more than 50 anesthesiologists) groups. The greatest proportion of DEs (48%) practiced in a medium-sized group. About 56% of DEs performed their own cases rather than mainly acting in supervisory roles. DEs were distributed about evenly across the Northeast (25%), Southeast (20%), Midwest (24%), and West (31%) of the U.S. Compared with those in large practices, DEs in small and medium practices were more likely to express concerns about professional encroachment by CRNAs, but were less likely to be concerned about suboptimal external perception of anesthesiologist value, especially when they reported often or exclusively performing their own cases. In large practices, those who often or exclusively performed their own cases were more likely to be concerned about compensation than those who mainly acted in supervisory roles. Among those who often or exclusively performed their own cases, those in medium and large practices were more likely to perceive threats to patient care than those in small practices. Practice location, defined by four U.S. geographical regions, significantly affected concerns about compensation only – anesthesiologists in the Southeast and West mentioned concerns about compensation more often than those in the Northeast.

**Table 5:** Perceived challenges to the profession of anesthesiology according to practice size and frequency of direct-entry anesthesiologists performing their own cases

	Count (%) of Direct-Entry Anesthesiologists’ responses (n = 463)					
Number of anesthesiologists in the group	Large (> 50)		Medium (10–50)		Small (< 10)	
Frequency of performing own cases	Never or Infrequently (n = 90)	Often or Exclusively (n = 73)	Never or Infrequently (n = 89)	Often or Exclusively (n = 132)	Never or Infrequently (n = 27)	Often or Exclusively (n = 52)
**Competition from non-physician providers**	35 (38.9)*	26 (35.6)*	**46 (51.7)**	**74 (56.1)**	**17 (63.0)**	**30 (57.7)**
**External perception of anesthesiologist value**	**38 (42.2)**	25 (34.2)	30 (33.7)	32 (24.2)*	8 (29.6)	12 (23.1)*
**Apathy from anesthesiologist colleagues**	**9 (10.0)**	4 (5.5)	2 (2.2)*	9 (6.8)	1 (3.7)	3 (5.8)
**Role of anesthesiologists**	4 (4.4)	2 (2.7)	2 (2.2)	3 (2.3)	0 (0.0)	2 (3.8)
**Compensation**	11 (12.2)*	**17 (23.3)**	17 (19.1)	23 (17.4)	3 (11.1)	8 (15.4)
**Uncertainty of changes in the healthcare system**	6 (6.7)	7 (9.6)	8 (9.0)	17 (12.9)	1 (3.7)	5 (9.6)
**Threats to patient care**	9 (10.0)	**10 (13.7)**	7 (7.9)	**18 (13.6)**	2 (7.4)	2 (3.8)*
**Corporationized management**	9 (10.0)	8 (11.0)	8 (9.0)	13 (9.8)	2 (7.4)	5 (9.6)
**Psychological pressures**	**9 (10.0)**	4 (5.5)	3 (3.4)*	12 (9.1)	2 (7.4)	1 (1.9)
**Meeting the standards**	1 (1.1)	0 (0.0)	1 (1.1)	3 (2.3)	1 (3.7)	3 (5.8)

Note: A bolded and underlined **figure** indicates that the number was statistically significantly different (after Bonferroni correction) from the number(s) with an asterisk in the same row

**Table 6:** Perceived challenges to the profession of anesthesiology according to practice location of direct-entry anesthesiologists

	Count (%) of Direct-Entry Anesthesiologists’ responses (n = 448^a^)			
	Northeast (n = 111)	Southeast (n = 91)	Midwest (n = 106)	West (n = 140)
**Competition from non-physician providers**	56 (50.5)	51 (56.0)	52 (49.1)	62 (44.3)
**External perception of anesthesiologist value**	31 (27.9)	26 (28.6)	35 (33.0)	48 (34.3)
**Apathy from anesthesiologist colleagues**	3 (2.7)	5 (5.5)	1 (0.9)	11 (7.9)
**Role of anesthesiologists**	1 (0.9)	2 (2.2)	5 (4.7)	4 (2.9)
**Compensation**	11 (9.9)*	**19 (20.9)**	15 (14.2)	**32 (22.9)**
**Uncertainty of changes in the healthcare system**	10 (9.0)	4 (4.4)	12 (11.3)	16 (11.4)
**Threats to patient care**	14 (12.6)	7 (7.7)	12 (11.3)	15 (10.7)
**Corporationized management**	9 (8.1)	12 (13.2)	10 (9.4)	12 (8.6)
**Psychological pressures**	13 (11.7)	2 (2.2)	7 (6.6)	8 (5.7)
**Meeting the standards**	1 (0.9)	1 (1.1)	3 (2.8)	4 (2.9)

^a^ 6 practitioners who chose “Not US” and 8 practitioners who chose “Other US” were not included in the table

Note: A bolded and underlined **figure** indicates that the number was statistically significantly different (after Bonferroni correction) from the number with an asterisk in the same row

## Discussion

A career in anesthesiology provides an opportunity for a varied, stimulating and fulfilling practice and has been popular as a specialty choice for graduating medical school students in the U.S. and elsewhere [11]. Residents in anesthesiology report satisfaction with their training [8], and many experienced anesthesiologists remain enthusiastically engaged in their work. Nevertheless, the profession of anesthesiology faces challenges and our survey study evaluated the perceptions of first-year graduates of U.S. anesthesiology residencies on this issue. The main finding was that these early career anesthesiologists’ perceived challenges fell into three broad themes - workforce competition from non-physician anesthesia providers and unease about external perception of anesthesiologist value, changes in the healthcare system that led to concerns about lower compensation and threats to patient care, and personal stressors including disquiet over burnout and the need to meet professional standards. These results highlight issues for programs and organizations to address. The perceived challenge to employment security posed by CRNAs and the perceived lack of appreciation for anesthesiologist value were most frequently cited. Although both AFs and DEs had similar concerns about the profession of anesthesiology, the relative weighting of their worries was different and may be the reasons behind – or a consequence of – their decision to pursue or not pursue a fellowship.

Demographic characteristics of AFs and DEs were similar and reflective of the life-stage of typical North American residency graduates. The influence of family factors on the decision to enter fellowship has previously been documented by Khan et al. among Canadian anesthesiology residents [6]. Having children may be a disincentive to fellowship because of the work hours involved, on-call responsibilities, and the unpredictability of these responsibilities. We suggest that implementation of measures to make fellowships more accommodating to anesthesiologists with, or intending to have, children would encourage more residents to consider that path [12]. Such measures might include enhancement of schedule flexibility, more accommodating leave-of-absence policies, support for nursing mothers, and improved access to childcare [13–15]. A greater amount of educational debt decreases the likelihood of a physician selecting a post-residency academic position and increases residency graduates’ interest in anesthesiology groups with an educational debt repayment program [3, 16, 17]. Although student debt was not reported as a major challenge in this study and debt burden was similar among AFs and DEs, those who chose to go directly into independent practice were slightly older, had more dependents, and were more likely to have a spouse who did not work outside the home, factors that may have influenced a perceived imperative to achieve financial security for their families. Although statistically significant, these differences were modest, and it is not clear that such modest differences would be determinative in making such an important life decision. Consistent with previous ﻿reports [7], a substantial proportion of respondents were not satisifed with their personal and professional life balance, although DEs expressed greater satisfaction than AFs.

From the perspective of U.S. anesthesiology residency graduates, the greatest challenge to the profession of anesthesiology identified from free-text comments was “competition from non-physician anesthesia providers”, the subject of more than half of all comments. This level of concern does not appear to be a new phenomenon [18, 19], but its persistence is striking. Of interest, compared to when these cohorts were CA-3 residents [2], AFs in training were slightly more concerned about this workforce competition while DEs were less concerned. We speculate that some DEs had seen first-hand how a highly functioning collaborative practice could work, whereas fellows lacked the “real world” experience and were apprehensive about their unknown post-fellowship employment. Providing more opportunities for fellows to participate in collaborative practice with advanced practice providers may help ease such concern and better prepare them for the care team they may lead in their future practice. Previous work has demonstrated the vulnerability and discrimination experienced by female anesthesiologists worldwide [20, 21]. Although many female DEs in our study were concerned about a perceived lack of differentiation between anesthesiologists and CRNAs (approximately 60% of CRNAs are female) [22], the proportion relative to other groups was not statistically significant. It was notable that those DEs who practiced predominantly in the “care team” model in a large practice (i.e., infrequently or never personally administered anesthesia as the sole provider) were more likely to raise a concern about the external perception of anesthesiologist value and a perceived lack of advocacy for the profession [23, 24]. To alleviate the concern, professional organizations and major hospitals could use diverse platforms and channels, including participation in medical conferences, strategic engagement on social media, and featured content in healthcare publications, to spotlight the contributions and expertise of anesthesiologists and foster a broader understanding and appreciation of their role in healthcare.

The choice of fellowship influenced the perception of competition from non-physician anesthesia providers. Advanced training was seen by some as a means to further differentiate anesthesiologists from non-physicians. Subspecialty training in either critical care medicine or cardiac anesthesiology was associated with a lower concern about workforce competition. Critical care medicine practice seems to be sufficiently different from operating room anesthesia that fellows feel assured that their physician subspecialty skills are more difficult to replace. Indeed, we previously documented that anesthesiology residents considered their critical care rotation as one of the most important rotations in clinical anesthesia training [8]. Although nurse practitioners increasingly deliver care in intensive care units, such individuals are usually not CRNAs. We also postulate that the routine integration of echocardiography training into cardiac anesthesiology fellowship helps differentiate the role of the cardiac anesthesiologists from that of the cardiac operating room CRNAs, which results in a decrease in the competition concern. Less easily explained, however, is that pain medicine fellows had the highest concern about workforce competition. Perhaps an explanation lies in the increasing number of non-anesthesiologist physicians and non-physicians who provide care in *pain management* [25, 26] in the U.S. and thus a heightened sensitivity to this issue among anesthesiology pain medicine fellows and consultants.

Within the second identified theme of healthcare system changes, concerns relating to financial compensation were most prominent. Many responses included specific concerns about decreasing reimbursements and bundled payments. Although female anesthesiologists’ salaries are 5–12% lower than those of male anesthesiologists [27] and female anesthesiologists face inequity in clinical practice [28], it was the male respondents in our cohort who were more likely to express concerns about compensation. DEs in large practices who often or exclusively perform their own cases were especially concerned about their renumeration. We speculate that their lack of a multi-room supervisory practice made these anesthesiologists feel vulnerable to identification as an “in-room provider”, similar to a CRNA or AA, with subsequent concern that they would be compensated at lower rates than those anesthesiologists whose practice model allows them to bill for simultaneous cases. Finally, our data, analyzed according to four U.S. regions of respondent practice location, demonstrated that compared to DEs in the northeastern region, those in the Southeast and West were more concerned about compensation. This may be partly due to regional differences in the anesthesiology workforce and the location-specific ratio of CRNAs to anesthesiologists [29, 30]. Although anesthesiologists are well compensated, our findings suggest that financial challenges are of significant concern at the outset of a career in the profession.

Although respondents had free range to identify any perceived challenges to the profession and to them, it is reassuring – and concerning - that primacy of patient welfare was highlighted as *the* principal challenge by about 8% of respondents. “Threats to patient care” were identified especially by those in the high acuity subspecialty of cardiac anesthesiology and those in large- and medium-sized groups who frequently or exclusively performed their own cases. One could speculate that this may be reflective of concerns held by those anesthesiologists who are routinely charged with caring for the most complex cases in what they perceive are increasingly corporate systems that prioritize economies and efficiencies.

The findings of our report are consistent with data obtained from senior anesthesiology residents as part of the ABA sequential cross-sectional survey study [2]. Similar themes were identified in that cohort, with work force competition from non-physician anesthesia providers being perceived as the greatest threat to the profession, followed by changes in the healthcare system and personal challenges. AFs, DEs, and senior anesthesiology residents were similarly concerned with undervaluation of anesthesiologists by others and lack of advocacy for physician values, an advocacy role more prominent than in any other medical specialty in the U.S.

As we have discussed in previous publications, our analyses based on repeated cross-sectional surveys are subject to limitations [2, 7, 8]. Of special relevance to the evaluation of perceived challenges to the profession was the potential for respondent bias, possible sources of which include subjective views of themselves, their practice, or the profession, and a deliberate portrayal of a specific view to the ABA. For example, respondents may have been reluctant to talk about their own compensation, but more willing to raise concerns about undervaluation of anesthesiologists. Additionally, although we strived to follow best practices of data collection and analysis, the free-text responses were open to interpretation, especially those that were brief and did not elaborate on the context. Our methodology allowed measurement of the frequency with which concerns were spontaneously expressed but not the prevalence of those concerns within the cohort. Respondents had to identify the *greatest* challenge facing the profession of anesthesiology; they would not likely have reported *all* challenges that may have been important to them. Further, our data reflect the views of U.S. anesthesiologists and were collected before the COVID-19 pandemic as part of a repeated cross-sectional study of stable cohorts, and do not reflect views of anesthesiologists outside of the U.S. or changes that may have occurred since the onset of the pandemic. Some of the post-pandemic changes in the U.S. include shortages of both anesthesiologists and CRNAs, upward compensation adjustments because of those shortages, and consolidation and increased corporatization of practices. Future studies could utilize the results of this study to make comparisons about how the challenges and perceptions have changed since the COVID-19 pandemic.

In summary, our data provide insight into the characteristics of AFs and DEs and their perception of challenges to the profession of anesthesiology in the U.S. The demographic characteristics of these two groups were largely similar. Although differences in age and family factors may suggest possible motivations for choosing fellowship or not, the importance of these small differences is uncertain. Our investigation of free-text responses to the question of the *greatest* challenge facing anesthesiology highlighted three major themes in descending order of frequency: workforce competition, healthcare system changes, and personal challenges. Members of the AF and DE groups shared these same concerns, but the relative weighting of these concerns was different and influenced by demographic and professional variables such as gender, fellowship subspecialty, and independent practice characteristics. These physicians represent the next generation of anesthesiologists in the U.S., who will drive the future directions of the specialty. We hope that our identification of the challenges they face and their concerns will inform advocacy and policies at programmatic and professional organizational levels.

### Electronic supplementary material

Below is the link to the electronic supplementary material.

Supplementary Material 1: Supplementary Table A

## Data Availability

The datasets generated and/or analyzed during the current study are not publicly available due to the confidentiality and sensitivity of the survey data and the stated terms with the survey respondents, but they can be made available from the Corresponding Author on reasonable requests.
