# Supplementary material for: Comparing characteristics and perspectives of U.S. anesthesiology fellows in training and anesthesiologists in their first year of practice
Source: BMC Med Educ. 2023 Dec 15;23:963. doi: 10.1186/s12909-023-04890-1 (PMC10725004; doi:10.1186/s12909-023-04890-1)
Supplement: Supplementary file 1 — Supplementary Material 1: Supplementary Table A [file 12909_2023_4890_MOESM1_ESM.docx]

Supplementary Table A. Examples of open-ended responses from anesthesiology fellows (AFs) and direct-entry anesthesiologists (DEs)

| **Category** | **Example Responses** |
| --- | --- |
| *Theme 1: Workforce Competition and Challenges* | |
| **Competition from non-physician providers** | “The advancement of the role of nurse anesthetists. I find that even in the workplace, they have more time to focus on advancing in administrative roles when anesthesiology physicians do not have the time or opportunities to make such career advancements…” (AF)  “The encroachment of CRNAs and AAs into the field of medicine and being allowed to practice independently without physician supervision. This appears to be driven by hospitals trying to reduce cost at the expense of patient care” (DE) |
| **External perception of anesthesiologist value** | “Educating the public about our expertise in physiology, pharmacology, and in the management of acute medical emergencies (in the operating room, emergency department and ICU) would help change public perception of anesthesiologists and our role in patient care and our role in health care overall.” (AF)  “It seems to be that surgeons are much more highly valued than anesthesiologists. I worry that CRNAs/AA are viewed just as valuable as anesthesiologists in both the public but also the medical system.” (DE) |
| **Apathy from anesthesiologists colleagues** | “…. Poor leadership especially among the more senior anesthesiologists. Looking out for oneself versus the younger anesthesiologists who are entering the workforce; mainly by selling out to larger national anesthesia companies.” (AF)  “Laziness on the part of physicians. If we are to continue to distinguish ourselves from others who 'provide' anesthesia, we must continue to advance the specialty and fill leadership roles in our hospitals and healthcare systems.” (DE) |
| **Role of anesthesiologists** | “Anesthesiologists must position themselves as invaluable perioperative physicians who are more than technicians or technical specialists, but well-rounded physicians in preoperative care, intensive care and post-op care as well as ultrasound for surgical patients.” (AF)  “maintaining and advocating for an anesthesia care team model in a way that is both safe, effective, efficient, has the best outcomes, maintains happiness amongst all members of the team, benefits patients and surgeons, without diluting ourselves and without losing sight of our value as a perioperative physician and leader.” (DE) |
| *Theme 2: Healthcare System Changes* | |
| **Compensation** | “Decreasing reimbursements increasing the amount of patients that you need to see to cover overhead expenses and thus decreasing the amount of time you have to spend with each patient.” (AF)  “Bundled payments affecting reimbursement and the perceptions of hospital administration of more cost-effective care with independent CRNA practice” (DE) |
| **Uncertainty of changes in the healthcare system** | “Politicization of health care, the removal of physicians from decision making both on a policy level as well as a medical individual patient level” (AF)  “The overall instability of healthcare in the US from political turmoil and the way that clinical practice is being altered by non-clinician administration, insurance, pharmaceutical and legal influences” (DE) |
| **Threats to patient care** | “Too many patients not enough support” (AF)  “Performing procedures on older, sicker patients in less time.” (DE) |
| **Corporationized management** | “Anesthesia management companies (AMCs) buying private anesthesia groups and demanding more work for less compensation are by far the largest threat to anesthesiology today. There needs to be a much larger organized effort to defend against these (often) hostile companies.” (AF)  “Big anesthesia corporations turning anesthesiologists into employees with no chance [of] working up the ladder.” (DE) |
| *Theme 3: Personal Challenges* | |
| **Psychological pressures** | “Systems want the same amount of work with less people which will lead to greater work hours and higher rate of burnout. Also when more anesthesiologist leave from said burnout mid-level providers will be plentiful to fill those positions” (AF)  “Increasing volume of cases and demands from the hospital (e.g. doing elective cases on Saturdays) which is making work-life balance more difficult.” (DE) |
| **Meeting the standards** | “Standards and certification requirements for anesthesiologist MD’s continue to increase - 2 written board exams, oral exam, now a new OSCE exam - meanwhile CRNA’s and AA’s, which have much less training and less stringent board requirements are able to perform more anesthesiologist level work” (AF)  “Too many exams and too much MOCA [*added note:* Maintenance of Certification in Anesthesiology]. Need to focus on ourselves and advocating for physicians overall and anesthesiologists for patient safety” (DE) |
